# Aseptic Meningitis With Multiple Cranial Nerve Symptoms Induced by Atezolizumab for Lung Adenocarcinoma: A Case Report

**DOI:** 10.7759/cureus.111696

**Published:** 2026-06-28

**Authors:** Satsuki Mikoshiba-Takahashi, Taiki Matsubayashi, Michiatsu Masuda, Kasumi Tsukamoto, Takashi Yamana, Mitsuhiro Kamimura

**Affiliations:** 1 Department of Respiratory Medicine, National Hospital Organization (NHO) Disaster Medical Center, Tokyo, JPN; 2 Department of Neurology, National Hospital Organization (NHO) Disaster Medical Center, Tokyo, JPN

**Keywords:** atezolizumab, immune check-point inhibitor, lung adenocarcinoma (adc), multiple cranial nerve palsies, neurological immune-related adverse events

## Abstract

We report a case of immune-related aseptic meningitis (irAE meningitis) presenting with multiple cranial nerve symptoms during immune checkpoint inhibitor therapy for lung adenocarcinoma. A 72-year-old woman diagnosed with left lower lobe lung adenocarcinoma initiated combination therapy consisting of carboplatin, paclitaxel, bevacizumab, and atezolizumab. Paclitaxel was subsequently discontinued due to peripheral neuropathy, and maintenance therapy with bevacizumab and atezolizumab followed. Eight months after starting treatment (after four cycles of atezolizumab maintenance), the patient presented with a three-week history of dysgeusia, anosmia, trismus, and neck pain. Cerebrospinal fluid (CSF) analysis revealed lymphocytic and monocytic pleocytosis, while cytological examination showed no malignant cells and bacterial cultures were negative. Brain and neck magnetic resonance imaging (MRI) showed no alternative etiologies. Intravenous prednisolone led to a rapid resolution of all symptoms. The patient was diagnosed with irAE meningitis with concomitant cranial nerve involvement, including the olfactory (I), trigeminal (V), facial (VII), and/or glossopharyngeal (IX) nerves. Intravenous prednisolone led to a rapid resolution of all symptoms. Although irAE meningitis is a known rare adverse event, cases complicated by multiple cranial nerve symptoms are extremely uncommon. This case highlights the phenotypic diversity of neurotoxicity associated with immune checkpoint inhibitors.

## Introduction

In recent lung cancer treatment, immune checkpoint inhibitors (ICIs) are widely used and play a central role. However, their use is associated with immune-related adverse events (irAEs) that can affect a wide range of organs [1-6]. Neurological irAEs (n-irAEs) are observed in approximately 1%-5% of patients receiving ICIs [6].

n-irAEs are broadly categorized into central nervous system (CNS) and peripheral nervous system (PNS) disorders. Within this framework, meningitis is typically classified as a CNS disorder, while cranial nerve palsy is considered a PNS manifestation [4,7].

Among n-irAEs, CNS involvement accounts for approximately 20%-30%, with meningitis occurring in about 3% of cases [8]. In clinical practice, aseptic meningitis generally describes cerebrospinal fluid (CSF) inflammation without evidence of a detectable bacterial or viral pathogen. Aseptic meningitis induced by ICIs presents with diverse neurological symptoms, including headache, altered consciousness, fever, seizures, and mental status changes, with significant variability in progression and severity [3,9]. The time to onset is also highly variable, ranging from a few days to several months after the initial administration [6,7,9]. Furthermore, irAE meningitis is frequently accompanied by other concurrent irAEs (36%) [6,10]. Because there are no disease-specific biomarkers for irAE meningitis, the diagnosis requires careful exclusion of other etiologies, including infectious meningitis, leptomeningeal metastasis, paraneoplastic neurological syndromes, and other autoimmune neurological disorders. Comprehensive evaluation using CSF analysis, microbiological studies, and neuroimaging is therefore essential for accurate diagnosis.

While a limited number of cases have described the coexistence of cranial nerve involvement and irAE meningitis, such presentations remain rare, and the underlying pathophysiology is not yet fully understood [5]. Therefore, we report this case of atezolizumab-induced aseptic meningitis complicated by multiple cranial nerve symptoms.

## Case presentation

The patient was a 72-year-old woman with a medical history of hypertension and hyperuricemia, and a 40-pack-year smoking history. Her family history was notable for gastric cancer in both her mother and her sister. Despite these first-degree relative occurrences, hereditary cancer syndromes were not strongly suspected based on the absence of early-onset or multiple primary malignancies. Consequently, further genetic evaluation was omitted as it was unlikely to impact the current management. She had a known allergy to house dust. Her Eastern Cooperative Oncology Group (ECOG) performance status was 1. A chest radiograph during a routine health checkup revealed a mass shadow in the left middle lung field (Figure 1).

**Figure 1:**
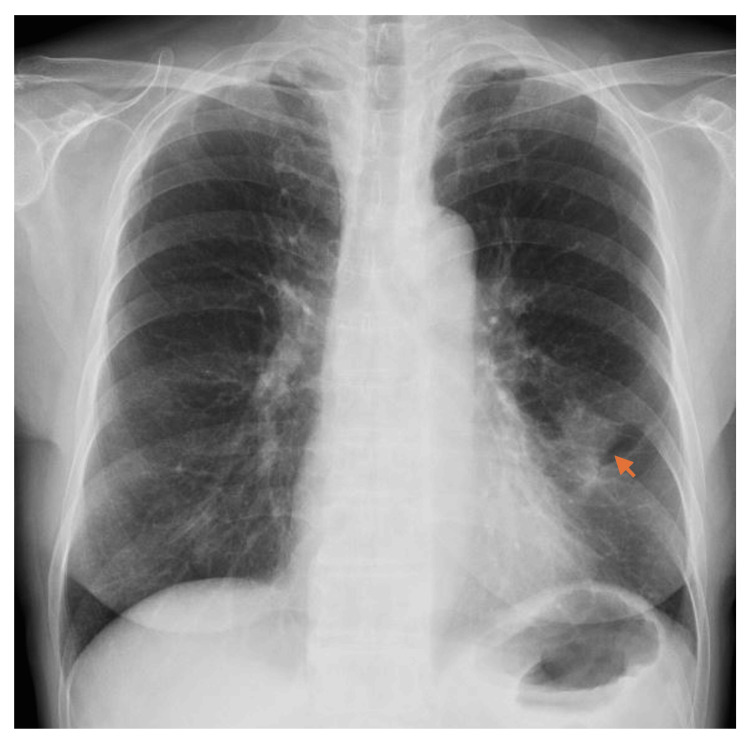
Chest radiograph showing a mass shadow in the left middle lung field Chest radiograph obtained during a routine health checkup demonstrating a mass shadow in the left middle lung field (arrow).

At initial presentation, chest and abdominal computed tomography (CT) demonstrated a left upper lobe mass with gastric lymph node enlargement (Figures 2A, 2B). Histopathological examination via bronchoscopy confirmed lung adenocarcinoma. Staging with contrast-enhanced brain magnetic resonance imaging (MRI) revealed a metastatic lesion in the left frontal lobe (Figure 2C). The patient was diagnosed with stage IVA (cT4N3M1b) lung adenocarcinoma.

**Figure 2:**
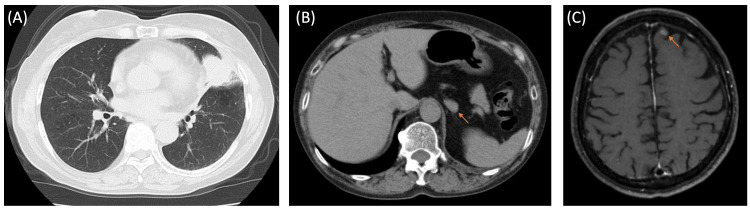
Radiological findings at initial diagnosis (A) Chest CT showing a mass in the left upper lobe. (B) Abdominal CT demonstrating metastatic enlargement of a gastrohepatic lymph node (arrow). (C) Gadolinium-enhanced brain MRI revealing a metastatic lesion in the left frontal lobe (arrow). CT: computed tomography; MRI: magnetic resonance imaging

Two months after the initial presentation, she commenced combination therapy with atezolizumab (1,200 mg every three weeks, intravenously), carboplatin (area under the concentration-time curve (AUC) 5) (300 mg/body), paclitaxel (200 mg/m²) (300 mg/body), and bevacizumab (15 mg/kg) (795 mg/body). After two cycles, a partial response (PR) was achieved. However, paclitaxel was discontinued due to Grade 3 peripheral neuropathy, and she transitioned to maintenance therapy with bevacizumab and atezolizumab according to the Common Terminology Criteria for Adverse Events (CTCAE) v5.0 [11]. Two months after treatment initiation, the patient developed Grade 3 irAE enteritis. Oral prednisolone 50 mg/day was initiated and tapered over seven weeks, leading to resolution without recurrence. Treatment was resumed five months after the initial start date. Seven months into treatment, she developed Grade 2 irAE hypothyroidism and began levothyroxine; atezolizumab and bevacizumab were temporarily withheld for observation.

Eight months after treatment initiation, the patient developed dysgeusia, anosmia, trismus, and neck pain, which gradually worsened over three weeks. On admission, the patient was alert and fully oriented. Body temperature was 36.8°C. Mental status examination revealed no cognitive impairment or disturbance of consciousness. Cranial nerve examination revealed anosmia, which strongly pointed to olfactory nerve (cranial nerve I) involvement. On clinical examination, trismus was noted, which was highly suggestive of trigeminal nerve (cranial nerve V) involvement; this was further supported by the patient's report of retro-orbital pain exacerbated by eye movement. Additionally, the patient reported dysgeusia, raising the possibility of involvement of the facial and/or glossopharyngeal nerves (cranial nerves VII and IX). No diplopia, facial weakness, dysarthria, hearing impairment, or dysphagia was observed. Motor examination demonstrated normal muscle strength in all extremities without focal weakness or paralysis. Sensory examination revealed pain extending from the right wrist to the dorsum of the hand without objective sensory loss. Deep tendon reflexes were preserved bilaterally without pathological reflexes. Coordination testing and gait assessment were unremarkable. Severe neck stiffness with restricted range of motion and a positive neck flexion test were observed, consistent with meningeal irritation. Laboratory investigations showed an elevated inflammatory response and a slight increase in tumor markers (sialyl Lewis X antigen (SLX) and carcinoembryonic antigen (CEA)). Autoantibody tests, including antinuclear antibodies and related serological markers, showed no significant findings. CSF analysis revealed pleocytosis with a mononuclear cell predominance and elevated protein levels. CSF cultures were negative, and cytology was negative for malignancy (Class II) (Table 1). Contrast-enhanced neck MRI and brain MRI showed no evidence of myositis, new metastases, or other alternative etiologies (Figures 3A, 3B).

**Table 1:** Laboratory parameters of serum and CSF on admission ACTH: adrenocorticotropic hormone; ALB: albumin; ALP (IFCC): alkaline phosphatase (International Federation of Clinical Chemistry); AMY: amylase; anti-AChR antibody: anti-acetylcholine receptor antibody; anti-GAD antibody: anti-glutamic acid decarboxylase antibody; anti-SSA antibody: anti-Sjögren’s syndrome-related antigen A antibody; anti-SSB antibody: anti-Sjögren’s syndrome-related antigen B antibody; BASO: basophils; BUN: blood urea nitrogen; Ca: calcium; CEA: carcinoembryonic antigen; CK: creatine kinase; Cl: chloride; CRE: creatinine; CRP: C-reactive protein; CSF: cerebrospinal fluid; D-dimer: fibrin degradation products; eGFR: estimated glomerular filtration rate; EOSINO: eosinophils; free T3: free triiodothyronine; free T4: free thyroxine; GOT (AST): glutamic oxaloacetic transaminase (aspartate aminotransferase); GPT (ALT): glutamic pyruvic transaminase (alanine aminotransferase); HGB: hemoglobin; IgA: immunoglobulin A; IgG: immunoglobulin G; IgM: immunoglobulin M; K: potassium; LDH (IFCC): lactate dehydrogenase (International Federation of Clinical Chemistry); LYMPH: lymphocytes; MONO: monocytes; Na: sodium; NEUTRO: neutrophils; P: phosphorus; pH: potential of hydrogen; PLT: platelet count; SLX: sialyl Lewis X antigen; T-BIL: total bilirubin; TSH: thyroid-stimulating hormone; UA: uric acid; WBC: white blood cell count

	Laboratory parameters	Value (units)	Reference range
Serum	WBC	9,200/μL	3,300-8,600
	HGB	11.6 g/dL	11.6-14.8
	PLT	40.7 × 10^4^/μL	15.8-34.8 × 10⁴
	NEUTRO	78.1%	40-70
	LYMPH	13.7%	20-45
	MONO	6.3%	2-10
	EOSINO	1.7%	1-5
	BASO	0.2%	0-1
	D-dimer	0.5 μg/mL	<1.0
	ALB	4.1 g/dL	4.1-5.1
	BUN	26.5 mg/dL	8-20
	CRE	1.22 mg/dL	0.46-0.82
	UA	5.9 mg/dL	2.6-5.5
	T-BIL	0.4 mg/dL	0.2-1.2
	GOT	14 U/L	13-30
	GPT	4 U/L	10-42
	ALP (IFCC)	83 U/L	38-113
	LDH (IFCC)	125 U/L	124-222
	AMY	276 U/L	37-125
	CK	19 U/L	41-153
	Na	135 mEq/L	138-145
	K	4.6 mEq/L	3.6-4.8
	Cl	94 mEq/L	101-108
	Ca	11.0 mg/dL	8.8-10.1
	P	4.5 mg/dL	2.5-4.5
	CRP	5.30 mg/dL	0.00-0.14
	Free T3	2.29 pg/mL	2.3-4.0
	Free T4	1.04 ng/dL	0.9-1.7
	TSH	12.1 μIU	0.5-5.0
	CEA	13.5 ng/mL	0-5.0
	SLX	55 U/mL	≤38
	Cortisol	24.0 μg/dL	6.24-18.0
	ACTH	60.3 pg/mL	7.2-63.5
	Anti-SSA antibody	Negative	Negative
	Anti-SSB antibody	Negative	Negative
	IgG	930 mg/dL	870-1700 mg/dL
	IgA	299 mg/dL	110-410 mg/dL
	IgM	86 mg/dL	33-190 mg/dL
	Anti-GAD antibody	<5.0 U/mL	<5.0 U/mL
	IgG	414.2 mg/dL	4.8-105 mg/dL
	Anti-AChR antibody	<0.2 nmol/L	<0.2 nmol/L
	Antinuclear antibody (ANA)	1:40	<1:40
CSF	CSF opening pressure	12.5 cmH₂O	6-25 cmH₂O (10-20 cmH₂O typical)
	CSF appearance	Colorless, transparent	Clear, colorless
	CSF total cell count	102	0-5 cells/μL (adults)
	Mononuclear cell count	100	40%-80% lymphocytes, 15%-45% monocytes
	Polymorphonuclear cell ratio	2%	0%-6%
	Mononuclear cell ratio	98%	94%-100%
	CSF protein	160.3 mg/dL	15-45 mg/dL
	CSF glucose	64 mg/dL	50-80 mg/dL (or 60%-70% of blood glucose)
	CSF LDH	48 U/L	<40 U/L (adults)
	CSF culture (bacterial)	Negative	Negative
	CSF culture (fungal)	Negative	Negative
	CSF cytology	Class II	Class I-II: benign, Class III-V: malignant

**Figure 3:**
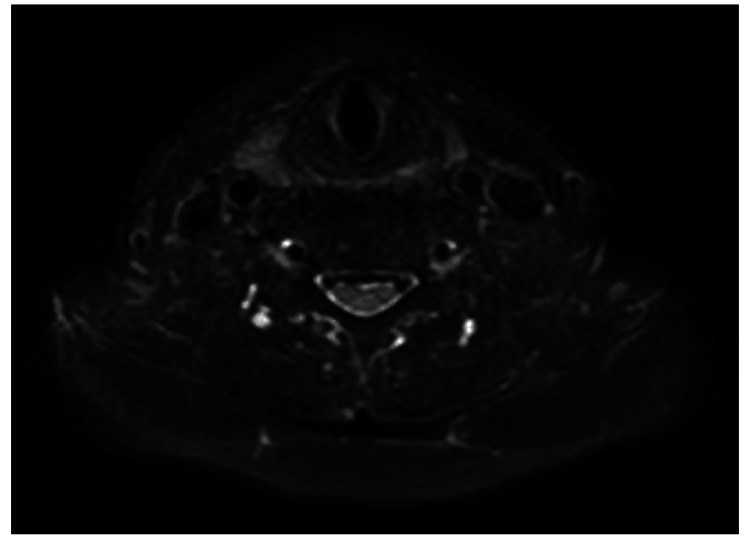
Cervical MRI findings on admission for irAE meningitis Short-tau inversion recovery (STIR) imaging revealing no inflammatory changes in the cervical muscles. MRI: magnetic resonance imaging; irAE: immune-related adverse event

Based on the clinical course, CSF findings, temporal association with ICI therapy, and the absence of findings strongly suggestive of infectious meningitis, the patient was considered to have irAE-associated aseptic meningitis complicated by multiple cranial nerve symptoms. Intravenous methylprednisolone (125 mg/day, equivalent to 2 mg/kg/day) was administered for seven days. The following day, her neck pain, anosmia, dysgeusia, and limb pain rapidly resolved. Given the prompt response to treatment, these findings were considered consistent with an irAE. The methylprednisolone dose was gradually tapered over nine months to a maintenance dose of 5 mg/day, with no recurrence of meningitis to date. Due to the risk of recurrent irAEs, neither atezolizumab nor cytotoxic chemotherapy has been resumed. The patient continues to be monitored through regular outpatient visits without any further systemic anticancer treatment. At the latest follow-up, six months after the onset of meningitis, the patient remained neurologically stable without recurrence of meningeal symptoms. Anosmia, dysgeusia, neck pain, and limb pain had completely resolved following corticosteroid therapy. Her ECOG performance status was 1, and she remained independent in activities of daily living. Serial CT and MRI examinations demonstrated sustained tumor control without progression.

## Discussion

irAE meningitis is diagnosed after the systematic exclusion of alternative etiologies, including infectious causes and carcinomatous meningitis, through MRI, lumbar puncture, and autoantibody screening [5,6,9]. In this case, despite elevated tumor markers, the absence of malignant cells in the CSF and the lack of new metastatic lesions on MRI ruled out carcinomatous meningitis. Although viral meningitis remained a differential diagnosis, the overall clinical and radiological findings were considered less consistent with an infectious etiology.

The clinical presentation of atezolizumab-related irAE meningitis typically includes fever, headache, and impaired consciousness [12]. While neck pain (a sign of meningeal irritation) is common, the combination of anosmia, dysgeusia, and trismus observed in our patient is highly atypical [1,13]. Due to the prominence of neck pain, we initially considered cervical spondylosis and myositis; however, these were subsequently excluded by MRI findings. The CSF findings in the present case, including lymphocytic pleocytosis and elevated protein levels, were consistent with those reported in previous cases [9]. Furthermore, the timing of onset in our case-after four cycles of atezolizumab despite an eight-month interruption in treatment-was broadly consistent with previous reports describing a median onset of two cycles (range: 1-14) [12].

The most striking feature of this case is the involvement of multiple cranial nerves. Although cranial neuropathy associated with ICIs has been documented, its coexistence with aseptic meningitis is exceptionally rare [2,14,15]. To the best of our knowledge, based on a literature search of PubMed as of February 2026, no cases of irAE meningitis accompanied by multiple cranial nerve symptoms have been reported. Previous reports of viral meningitis and encephalitis have suggested that inflammatory spread from the meninges to the cranial nerve roots may contribute to multiple cranial nerve symptoms [15]. These observations suggest that the underlying pathophysiological mechanism may involve extension of inflammation from the meninges to the cranial nerve roots. As a limitation of the present case, the assessment of cranial nerve involvement was partly based on the patient's subjective symptoms. Specifically, trigeminal nerve involvement was evaluated on the basis of impaired mouth opening observed during the clinical examination, whereas olfactory, facial, and glossopharyngeal nerve involvement was assessed primarily from the patient's reported symptoms. Therefore, the localization and extent of cranial nerve involvement remain partly presumptive. Nevertheless, the combination of these symptoms with lymphocytic pleocytosis, elevated CSF protein, negative infectious studies, and corticosteroid responsiveness supported the diagnosis of ICI-related aseptic meningitis with possible cranial nerve involvement. In the present case, the absence of sensory disturbances in the limbs or abnormalities in deep tendon reflexes argues against a concurrent systemic immune-mediated peripheral neuropathy. Instead, the clinical constellation raised the possibility of inflammatory involvement of the cranial nerve roots associated with meningitis. Furthermore, the rapid resolution of symptoms following high-dose corticosteroid therapy was consistent with a reversible inflammatory process, supporting the possibility of irAE-associated aseptic meningitis.

A comprehensive paraneoplastic antibody panel (including anti-Hu, Yo, Ri, Ma2, and CV2 antibodies) and viral PCR testing of the CSF were not performed, which represents a limitation of this report. Repeat CSF examination was also not performed because the patient showed rapid clinical improvement after corticosteroid therapy. However, several findings strongly favored irAE meningitis, including the absence of fever, lack of radiological evidence of infection or leptomeningeal dissemination, coexistence of multi-organ irAEs, and prompt responsiveness to corticosteroid treatment.

Finally, our patient had a history of irAE enteritis and hypothyroidism, suggesting a possible predisposition to multi-organ irAEs. The decision to resume ICIs in such patients must be made with extreme caution, balancing the severity of neurological toxicities against the necessity of oncological control. When ICI rechallenge is not feasible, alternative treatment strategies such as chemotherapy, targeted therapy, or supportive care should be considered according to the patient’s clinical condition and oncological status.

## Conclusions

We reported a rare case of atezolizumab-induced aseptic meningitis characterized by multiple cranial nerve symptoms, including anosmia, dysgeusia, and trismus. This case highlights the clinical diversity of n-irAEs and suggests that meningeal inflammation can extend to the cranial nerve roots. Clinicians should maintain a high index of suspicion for irAE meningitis in patients treated with ICIs who present with atypical neurological symptoms. Early diagnosis and prompt corticosteroid therapy are crucial for favorable outcomes, particularly in patients with a history of recurrent irAEs.
